# A Novel Method for Assessing the Chaperone Activity of Proteins

**DOI:** 10.1371/journal.pone.0161970

**Published:** 2016-08-26

**Authors:** Nevena Hristozova, Peter Tompa, Denes Kovacs

**Affiliations:** 1 Structural Biology Department, Flemish Institute of Biotechnology, Brussels, Belgium; 2 Structural Biology Department, Free University Brussels, Brussels, Belgium; 3 Institute of Enzymology, Hungarian Academy of Sciences, Budapest, Hungary; University of Pittsburgh, UNITED STATES

## Abstract

Protein chaperones are molecular machines which function both during homeostasis and stress conditions in all living organisms. Depending on their specific function, molecular chaperones are involved in a plethora of cellular processes by playing key roles in nascent protein chain folding, transport and quality control. Among stress protein families–molecules expressed during adverse conditions, infection, and diseases–chaperones are highly abundant. Their molecular functions range from stabilizing stress-susceptible molecules and membranes to assisting the refolding of stress-damaged proteins, thereby acting as protective barriers against cellular damage. Here we propose a novel technique to test and measure the capability for protective activity of known and putative chaperones in a semi-high throughput manner on a plate reader. The current state of the art does not allow the *in vitro* measurements of chaperone activity in a highly parallel manner with high accuracy or high reproducibility, thus we believe that the method we report will be of significant benefit in this direction. The use of this method may lead to a considerable increase in the number of experimentally verified proteins with such functions, and may also allow the dissection of their molecular mechanism for a better understanding of their function.

## Introduction

Molecular chaperones are a diverse group of proteins that play critical roles in assisting the folding and assembly of nascent protein chains [1], refolding proteins, assisting protein translocation through membranes [2], and often facilitating protein degradation [3]. Besides physiological processes, these helper proteins also fulfil crucial functions under unfavorable environmental conditions, such as temperature or osmotic stresses, but also in disease conditions and in response to a pathogen attack [3]. Chaperones are involved in the molecular response to stress in plants [4], bacteria [5], and animals [6], and were identified as key players in host-pathogen interaction and in the formation of innate and adaptive immunity [7,8]. During unfavorable environmental conditions, infection or disease, animals rely on a wide variety of chaperones to prevent and reduce the damage caused to cellular compartments and molecules [9]. Overexpression of chaperones in flies and mice so far proved effective in suppressing neurodegeneration [10,11]. In some cardiovascular diseases, cataract, alcoholic hepatitis, cystic fibrosis, phenylketonuria, and in a range of human cancers the protein homeostasis machinery was also shown to be involved as the first line of defense against the disease, thus rendering it a molecular marker for diagnostics [12,13,14]. This makes proteins with chaperone function potential drug targets, due to their function in various pathological and physiological processes inside the cell. Therefore functional and mechanistic characterization is crucial.

The current state of the art in chaperone identification and discovery combines *in silico* homology screens [14], analysis of transcriptomics data [15], and co-purification with other proteins [16]. Such methods are effective in pooling proteins with stress-related function, but they have limited reproducibility and inherent high error rates of false positives, and require detailed *in vitro* studies for verification [17]. Methods for chaperone activity testing are under patenting (e.g. application number PCT/US2012/037131), nonetheless, the lack of fast and reliable high-throughput methods for assessing the protective capacity of proteins considerably hinders the discovery and characterization of novel proteins with such functionality. Thus, we propose a semi-high throughput *in vitro* method for the fast and reliable assessment of chaperone capacity, based on measuring enzyme activity in a 96-well plate format, where the protective activity of a putative chaperone is defined by the retention of enzyme activity during adverse conditions. The proposed method will be of use when a high number of proteins or conditions need to be screened, efficient concentration screens (efficacy) are to be done, or extensive mutation analysis needs to be carried out, for example. In order to show the use of such a method (i.e. to provide proof of principle), we have selected four widely used substrates from the chaperone field: citrate synthase [18], luciferase [19], lactate dehydrogenase [20] and alcohol dehydrogenase [21], optimized the enzymatic assays for the plate reader format and set up the chaperone assay within the plate. We then designed a configuration of the measurements that provides adequate statistical power, and enables us to compare the activity of various enzyme-chaperone mixtures.

In addition to testing the technique on well-established chaperones, we demonstrate the use of the proposed method by characterizing the putative chaperone ERD10. The Early Response to Dehydration protein 10 is a Late Embryogenesis Protein (LEA), described as a dehydrin [22]. Dehydrins are plant proteins primarily involved in cellular and systemic responses to drought stress via various, not fully understood, mechanisms [23]. Kovacs et al. recently explored the putative proteostatic activity of ERD10 and provided formal proof that ERD10 can prevent the aggregation of a range of proteins [24]. Thus, we aim to further explore the protective effect of dehydrins, also demonstrating the advantages of the new technique.

We also saw the potential of this technique in measuring the protective activity of complex systems, such as whole-cell protein extracts. *In vitro* experiments traditionally dominated the field of measuring the chaperone activity of purified proteins. Recently, the proteostatic activity of proteins is also studied *in vivo*, via overexpression in cell lines, and follow its effect via phenotype screens (i.e. stress resistance), addressing their subcellular localization, interaction with other proteins or organelles, and protection of cellular proteins [25–27]. We propose to measure the protein activity protection of a total protein extract from cells grown under physiological and stress conditions when endogenous chaperones are being overexpressed. One of the most prominent ways to discover stress-related proteins is by microarrays. In eukaryotes subjected to stress, the first and highest scoring genes to be expressed and respectively identified, are stress-related proteins and signaling kinases [28]. Bacteria, like other organisms, also express a number of chaperones even when they are not subjected to environmental stress [29]. Our assay is expected to quantify the chaperone activity of the complete protein machinery in a comparative manner.

## Materials and Methods

### Expression and purification of ERD10

ERD10 was cloned, expressed and purified as described in Kovacs et al [24]. After obtaining pure protein (as analyzed via SDS-PAGE electrophoresis and mass-spectrometry, ERD10 sample was lyophilized and subjected to elemental analysis for quantification in the Department of Analytical chemistry in the Vrije Universiteit Brussel.

### Activity measurements

In order to study the chaperone activity of a protein, we developed a set of fast, reliable and inexpensive protocols. They are all based on standard enzymatic reactions and the ability of the putative chaperone to protect enzyme activity under stress conditions. We optimized these protocols for four standard enzymes, widely distributed among different species–Alcohol dehydrogenase (ADH, EC 1.1.1.1), Citrate synthase (CS, EC 2.3.3.1), Lactate dehydrogenase (LDH, 1.1.1.27), and Luciferase (Luc, EC 1.13.12.7). We altered and optimized the protocols of enzyme activity measurement to fit the format of a microplate reader [30–34].

#### Comparative definition of enzyme activity

All the protocols follow spectrophotometric reading after the start of the enzymatic reaction. The measurements were performed in a Biotek Synergy Mx microplate reader at wavelengths characteristic for the reactions, until a stable plateau was reached. In all cases, the activity of the corresponding enzyme was defined by the slope of the initial, linear phase of the curve. 100% activity was set to be the activity of the enzyme prior to stress and without the addition of chaperones.

#### Alcohol dehydrogenase activity

ADH catalyzes the oxidation of alcohols to aldehyde or ketone using NAD^+^ as proton acceptor. The reaction leads to production of NADH, which can be followed at 340nm. The reaction was performed using 0.1 μM yeast ADH (A7011, Sigma), 6mM NAD^+^ (N7004, Sigma), and 3.75mM EtOH in 100mM TrisHCl, supplemented with 250mM NaCl, at pH 8.8 [34]. Data collection started immediately after the addition of NAD^+^ and EtOH to the enzyme solution and was continued for 3 minutes at RT to obtain the initial part of the activity curves.

#### Citrate synthase activity

The activity of citrate synthase was measured based on the method described by Srere [33]. Porcine citrate synthase is the first enzyme of the citric acid cycle and exists in almost all cell and tissue types. It converts Acetyl-CoA and oxaloacetate into citrate and releases reduced coenzyme-A. The latter reacts with the Ellman’s reagent (DTNB) enabling the reaction to be followed at 412 nm where reacted DTNB absorbs. The reaction was performed with 6 nM citrate synthase (C3260, Sigma), 0.45 mM Acetyl-coA (A2056, Sigma), 0.5 mM oxaloacetate (O4126, Sigma), and 0.1 mM DTNB (D8130, Sigma) in 50 mM Tris-HCl buffer supplemented with 250mM NaCl, at pH 7.5, and followed for 3 min at 30°C.

#### Lactate dehydrogenase activity

LDH is present in almost all cell types and organisms; it catalyzes the reversible conversion of pyruvate to lactate. The enzyme uses NADH cofactor and oxidizes it to NAD^+^. The reaction solution contained 10μM bacterial LDH (L3888, Sigma), 6.6 mM NADH (AE12.1, Carl Roth), and 30 mM Sodium pyruvate (8793.1, Carl Roth), in 200mM Tris-HCl, at pH 7.3 [35]. The reaction was followed by monitoring the decrease in absorbance at 340 nm for 3 min at RT.

#### Luciferase activity

The light-producing enzyme luciferase is present in various bioluminescent organisms, where it can have a true respiratory function when the major protein components of the respiratory pathway are impaired [36]. The enzymatic activity was followed in 1mM PBS, at pH 6.8 and RT at following the concentrations: 0.5μM firefly luciferase (L9506, Sigma), 1.3 mM NADH (AE12.1, Carl Roth), 0.042 mM FMN (F2253, Sigma), and 0.0025% decanal (D7384, Sigma) [37]. The enzyme activity was defined by the amount of the emitted light, recorded in a luminometric experiment in a plate reader with 1s integration time and at identical gain settings between the different samples.

### Heat-induced inactivation

Once the activities of the enzymes were recorded, another aliquot of the same batch was subjected to high-temperature stress in a thermo block (PST60HL, Boeco). We have tested various ways for enzyme deactivation, and found that without a direct contact with the heating unit, the level of inactivation is not even due to a temperature gradient across the plate. The heating block used in these experiments transfers the heat via a direct contact with the plate, which is further enhanced by continuous mild shaking (250 rpm). The temperatures and times optimized for deactivation in the given setup were first selected from literature and were further optimized as to limit evaporation and increase reproducibility. Therefore, enzymes were deactivated at following temperatures: ADH at 46°C for 60min; CS at 44°C for 40min; LDH at 56°C for 60min, Luc at 44° for 30min.

As the reaction volumes are rather small, even limited evaporation would lead to a change in the reagent concentrations in the solution and compromise the measurements. Thus, we sealed the plates at the heating step with a VIEWseal thermo-resistant microtiter plate sealing foil (676070, Greiner Bio-One). Co-factors and reporter agents (where applicable) were added just before the measurement to avoid thermal decomposition. Enzyme activities have always been compared between different samples e.g. before and after stress; in the presence or absence of a putative chaperone; at various concentrations; different strength and/or duration of the stress condition. As a proof of concept, we used well-known molecular chaperones, reported to be effective against temperature deactivation, among other types of stress. Namely, we used GroEL, Hsp70, and Hsp90 in equimolar ratios or in excess to the enzyme (client) [14,38–45].

### Chaperone assay with CS in the presence of ERD10

We used the CS activity assay described above to study the protective effect of ERD10 against thermal deactivation of the enzyme. Lyophilized ERD10 was dissolved in 50 mM aqueous solution of 50mM Tris-HCl, 250 mM NaCl at pH 7.5, and added to the CS sample in different ratios–from equimolar to a great excess of ERD10 as compared to CS. Activity of CS was measured as described above, before and after the protein mix was subjected to stress at 44°C for 40 min. The enzymatic activity was compared between samples with and without the addition of ERD10, and between samples with different concentrations of ERD10.

### Total protein extract preparation

To prepare total protein extracts, we used non-transformed *E*. *coli* BL21 (DE3) cells strains. The cells were grown on full LB media at 37°C with shaking, until OD_600_ = 1. After reaching the required optical density, the cells were transferred to 42°C and aliquots were removed before stress and after 3 hours of incubation at high temperature. The samples were then centrifuged at 13000 rpm at 4°C in a bench-top centrifuge, for 1h to pellet the cells. The pelleted cells were resuspended in a lysis buffer containing 50mM Tris-HCl, 50mM NaCl, and 5mM DTT at pH 7.5, supplemented with protease inhibitors, DNAse (50μg/ml) and were lysed by sonication (10 pulses/10s each, followed by 30s on ice). The lysate was loaded into a 3kDa cut-off dialysis membrane, and dialyzed against a buffer of 50mM Tris-HCl, 50 mM NaCl at pH 7.5 buffer, supplemented with protease inhibitors overnight. The total protein extract was quantified using the Bradford assay method (B6916, Sigma).

### Chaperone assay in the presence of total protein extract from *E*. *coli*

We used the CS activity assay described above to study the protective effect of total protein extracts from *E*.*coli* against thermal deactivation of the enzyme. Final amount of 0.02 mg of protein extract per reaction was added to the CS assay instead of a purified chaperone. In order to anticipate the increased background activity of cell extracts, we also added the total protein extract to a blank reaction mix (containing substrates and reporter agent, but no CS). The assay was further performed as already described above, before and after subjecting the protein mix to stress at 44°C for 40 min. The enzymatic activity of CS was compared between samples with and without the addition of total *E*.*coli* protein extract.

### Data analysis and representation

All assays were performed with replicates, ranging from 5 to 12 replicate per experimental condition to allow robust statistical analysis. The slopes of each reaction were calculated using GraphPad Prism ver.6 and were subsequently normalized to the activity of the corresponding enzyme before it was subjected to stress. For each sample, mean and 95% confidence interval was calculated before normalization. Normalized values were plotted as curves to illustrate the gradual deactivation of each enzyme. The statistical significance of the differences between samples was calculated via one-way ANOVA and t-test.

## Results

Chaperone assays are widely used to assess the putative function of proteins being expressed or overexpressed in the cell under stress conditions, such as high temperature, oxidative stress, high salinity, UV, etc. The chaperone activity of a protein is determined by the protection of a client protein either against aggregation or the loss of activity evoked by stress. Although chaperone assays are widely used, they usually require tedious experiments due to the high standard deviation of each measurement mostly attributed to experimental, and not biological, error. Classical measurements usually involve the withdrawal of an aliquot from an enzyme assay, followed by a subsequent evaluation of specific properties of the sample and comparison of results between assays with and without the chaperone. Such assays are very sensitive to changes in concentration, to the uniformity of stress applied on the sample and also to other experimental parameters such as time passed between mixing and start of photometric detection. Accordingly, to reduce experimental error of such an assay, both the number of technical replicates and the accuracy of the assay has to be increased.

To this end, we aimed to transfer activity assays to a plate reader, thereby increasing the number of parallel samples and also the reliability of measurements by keeping hands-on time low. We aimed to utilize enzyme activity assays commonly used in the field. As a result, we report the activity assays of four standard enzymes on a plate reader, following their heat-induced deactivation. To demonstrate the efficacy of the plate reader format, we have tested the protective activity of HSP90, HSP70 and GroEL/ES on CS as substrate.

### Activity assays on a plate reader

**Fig 1:**
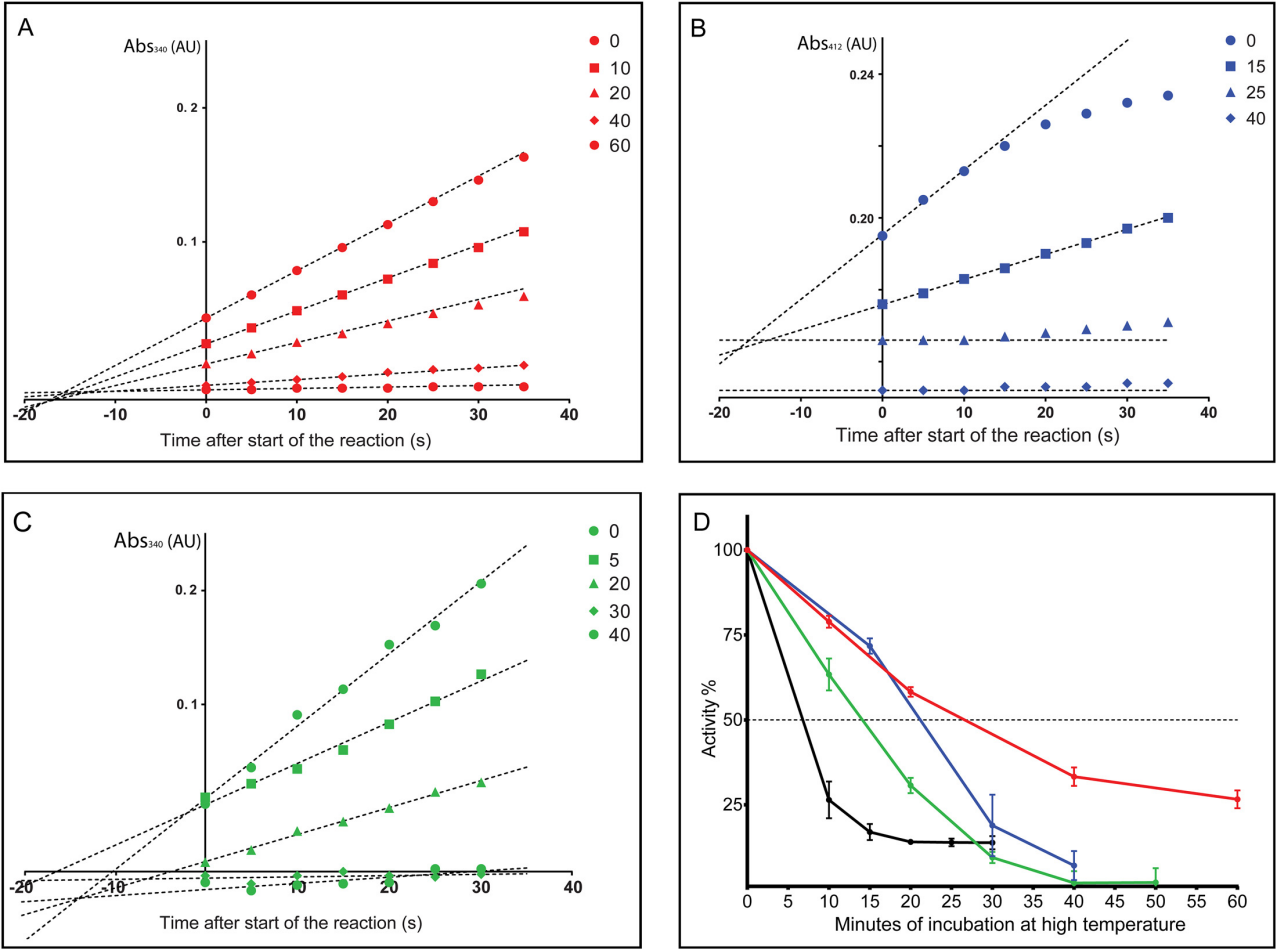
Activity of ADH (A), CS (B), LDH (C), and Luc (in black on panel D). The activity of each enzyme was recorded at the specific wavelength for each reaction. The different data points on panels (A), (B) and (C) represent the raw data readout from each time-point during the deactivation by high temperature. The lines represent the slopes calculated for the initial linear phase of each reaction. Due to the necessary mixing step in our protocol we miss the first few seconds of the reaction, thus we extrapolated the slopes to time zero of each reaction. The observed activity of the enzymes drop as shown by the decreasing slopes as a function of time at high temperature. (D) The deactivation of each enzyme was shown as % relative remaining activity: ADH (red), CS (blue), LDH (green), and Luc (black). The data for each enzyme was normalized to the activity of the non-stressed enzyme. The data was plotted as mean and 95% CI.

Activity of an enzyme solution is defined by the initial, maximum velocity of a reaction, measured at 10-fold excess of the substrate over the K_m_. Under such conditions, the measured reaction velocity is above 90% of the V_max_, and remains linear over a longer period of time, due to the large excess of substrate over the product. In practice, the velocity is measured as the slope of the initial linear phase of the reaction (Fig 1). For comparison of different samples, it is important to define the velocity exactly at the same time point after mixing the components, in order to have an accurate assessment of the effect of the chaperone. Nonetheless, due to technical limitations, the first few seconds of the reaction cannot be observed, which is why the slopes of the curves converge at the negative side of the timeline. The point where the fitted slopes cross shows that a linear fit has been done on the linear phase of the reaction curve. The slopes can then be compared. It is also apparent from Fig 1 that the experimental setup is suitable to measure changes in the velocity (see next section). Confidence intervals of technical repeats illustrate the accuracy of the measurements.

### Heat-induced inactivation of enzymes

**Fig 2:**
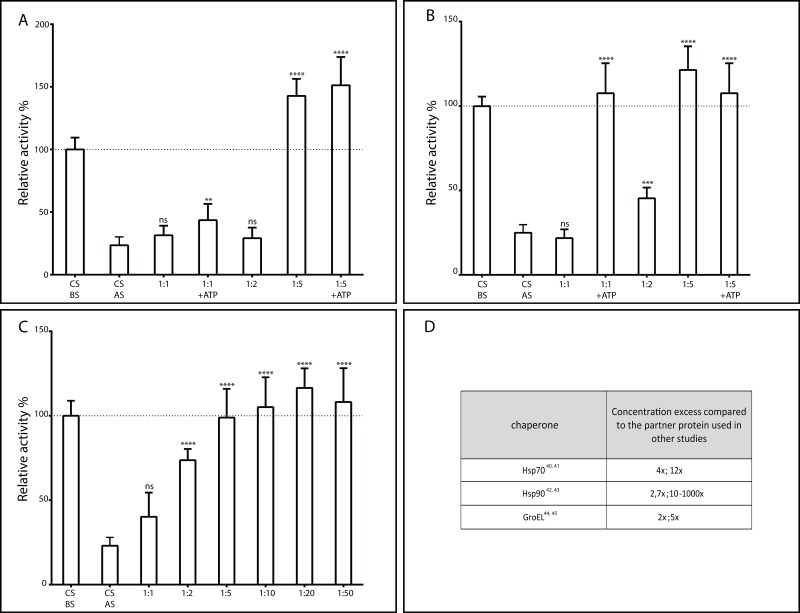
Hsp70 (A), Hsp90 (B), and GroEL (C) protect citrate synthase against temperature deactivation. The activity of the enzyme after temperature stress alone (CS AS) or in the presence of different ratios of the respective chaperone (and excess ATP where noted) was measured as described in the Materials and Methods section, and plotted on each panel. The bars represent the mean of the parallel measurements; the error bars indicate the 95% confidence intervals of each measurement. The data was normalized to the activity of the enzyme alone before it was subjected to temperature stress (CS BS). One-way ANOVA analysis was performed in each case to assess the significance of the differences of the protection effect of each chaperone compared to the CS AS. Panel D shows examples of the concentrations of chaperones used in previous experiments to assess protective activity.

Once we optimized the enzymatic assays for a plate reader, we checked whether these assays would be suitable for a chaperone activity assay. To this end, we applied high temperature stress as described in Materials and Methods and compared activities before and after stress. To ensure sufficient dynamic range for the assays, we have optimized the temperature and the time of incubation so that a drop in activity to about 20% of the original activity occurs (Fig 1D and Table 1). The kinetics of deactivation of the different enzymes are different, thus optimal temperatures and times differ as follows: ADH, 46°C for 60 min; CS, 44°C for 40 min; LDH, 56°C for 60 min, and Luc, 44° for 30 min (Fig 1). For example, the activity of CS was observed to drop from 100% to under 20%, in 40 minutes at 44°C. After repeating the deactivation of each enzyme several times and measuring several points along the deactivation, we concluded that measuring two data points is sufficient to make a conclusive comparison of initial activity and activity after deactivation (for details, view deactivation kinetics of the respective enzyme, Figs 1 and 2). Fig 1D shows a decrease of activity as a function of time upon high-temperature stress. While the activity of LDH drops below 10% in one hour at 56°C, the activity of ADH drops to about 25% in the same time at 43°C. Luciferase activity, unlike that of ADH, CS and LDH, is measured by recording emitted light, which is then converted to relative activity. Luciferase is well known for being temperature sensitive, thus it loses 75% of its initial activity in 10 minutes at 44°C. The corresponding temperatures and incubation times for each enzyme were selected so that the deactivation can be observed in a relatively short time, without inducing an immediate aggregation of the proteins (Table 1). Nonetheless, we aimed to achieve a significant deactivation of the enzymes without prolonged exposure to high temperatures to avoid evaporation, which would lead to an increase in error due to concentration changes of the reagents in each reaction, rendering comparisons impossible. Each measurement was performed multiple times (at least 6, at most 12 technical repeats), so a statistical evaluation of the data can be performed by calculating the 95% confidence interval, and running an one-way ANOVA tests. 95% CI in combination with hypothesis testing is a powerful statistical test for showing differences between two sample populations.

**Table 1:** Loss of activity of each enzyme as a function of time at high temperature.

ADH			CS			LDH			Luc		
Time (min)	Average	SD	Time (min)	Average	SD	Time (min)	Average	SD	Time (min)	Average	SD
0	100	7.72	0	100	3.67	0	100	5.27	0	100	19.69
10	78.88	6.29	25	60.71	14.22	10	66.44	4.95	10	26.45	5.42
20	58.25	3.57	35	18.88	9.17	20	32.17	2.37	15	16.99	2.36
30	42.71	1.51	45	7.14	4.38	30	9.94	1.74	20	14.07	0.17
40	33.12	0.72				40	2.08	3.66	25	13.94	1.14
50	26.50	0.95				50	2.23	4.44	30	13.86	1.94
60	21.68	0.60									

### Chaperones protect enzymes against activity loss

Once the enzyme assays were optimized, we tested whether such assays are suitable to measure the protective activity of known chaperones. To this end, we have selected three well-known chaperones from the literature, HSP70 [46], HSP90 [39], and the GroEL/GroES chaperonin complex [47]. According to previous publications, the selected chaperones usually show protective effect between 1:1 and 1:5 concentration ratios (Fig 2). We have measured the activity of CS at the same concentration ratios before and after temperature stress, and found that at 1:1 ratio it shows a rather limited protective effect in all the three cases: CS in the presence of GroEL retains about 30% of its original activity; while at equimolar concentrations of Hsp70 and Hsp90, it is not protected at all (Fig 2). The protective effect increases significantly at 1:2 molar ratio, where two-fold molar excess of GroEL retains the initial CS activity completely; whereas 47% and 30% of the enzymatic activity is retained in the presence of Hsp90 and Hsp70 respectively. At 1:5 ratio, Hsp90 can protect CS against temperature inactivation completely in our assay (Fig 2).

We also compared the efficacy of Hsp70 and Hsp90 in the absence and presence of ATP (Fig 2A and 2B). We found that the addition of ATP increased the protection of the enzyme at 1:1 ratio in both the cases of Hsp70 and Hsp90, but did not at a 5 times excess of the chaperone.

### Chaperone functions of ERD10

**Fig 3:**
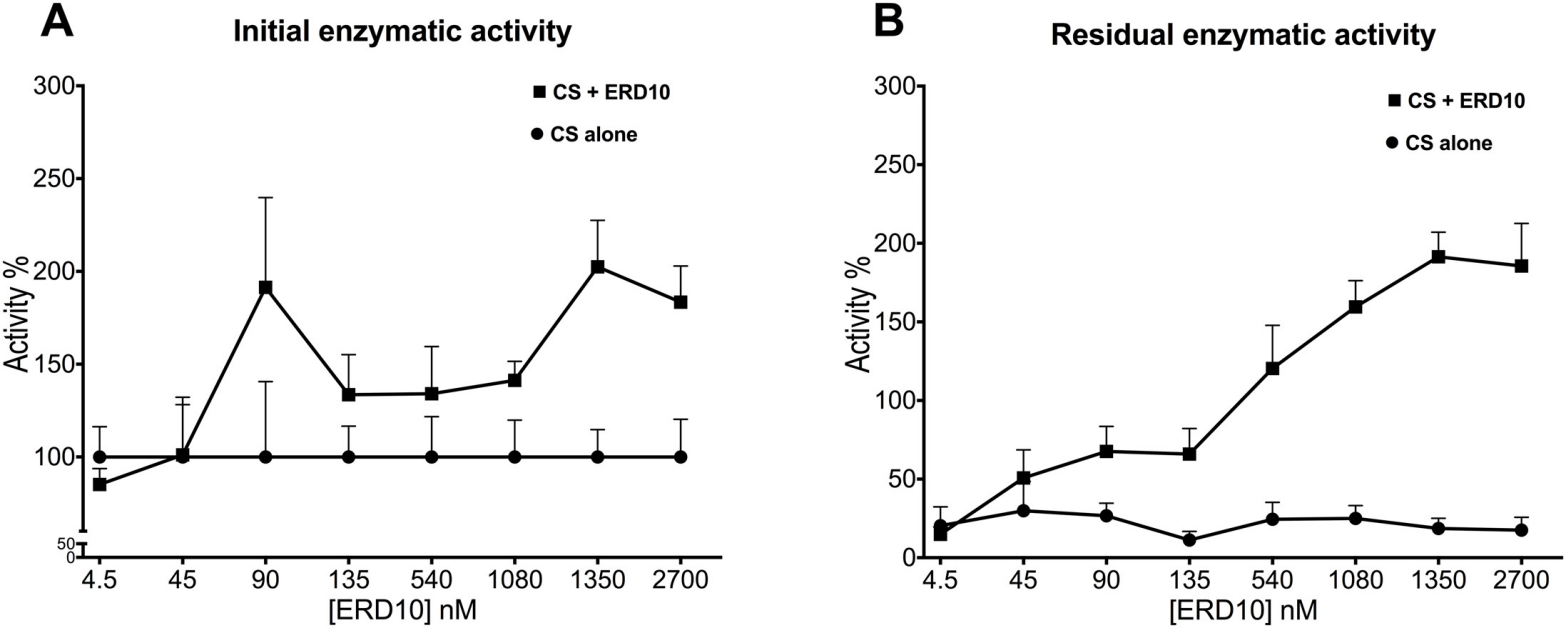
Enzymatic activity of CS is protected by ERD10 against temperature deactivation. The activity of CS was recorded before (A) and after stress (B) of 44°C for 40min in the presence (■) and absence (•) of different concentration of ERD10 as described in the Material and Methods section. Activity is presented as percentage of the activity of the non-stressed CS without the addition of ERD10. Concentration of CS is constant at 4.5nM. The symbols represent the mean, and the error flags—the 95%CI.

ERD10 was already shown to have a protective effect against protein aggregation by Kovacs et al [24]. In this work, we also formally demonstrated the protective effect of ERD10 on enzyme activity, apart from the protection against aggregation. The protective effect of ERD10 on CS activity was measured at various molar ratios which were then compared to the control reaction where CS was alone (Fig 3). After 40min at 44°C, CS alone loses around 80% of its activity, while this is not the case when excess of ERD10 is added to the sample: in the presence of 20x molar excess of ERD10, CS retains its activity completely. This protective effect of the dehydrin increases with increasing concentrations, as shown in Fig 3. The apparent increase of activity of CS above 100% in the presence of ERD10 is believed to be due to reactivation of inactive species in the commercial enzyme product, which appeared to be homogeneous even when subjected to size-exclusion chromatography, apparently devoid of unfolded, misfolded or aggregated species. This phenomenon deserves further investigation as it has been observed in several other experiments with chaperones [48,49].

### Chaperone capacity of total protein extract from *E*. *coli*

**Fig 4:**
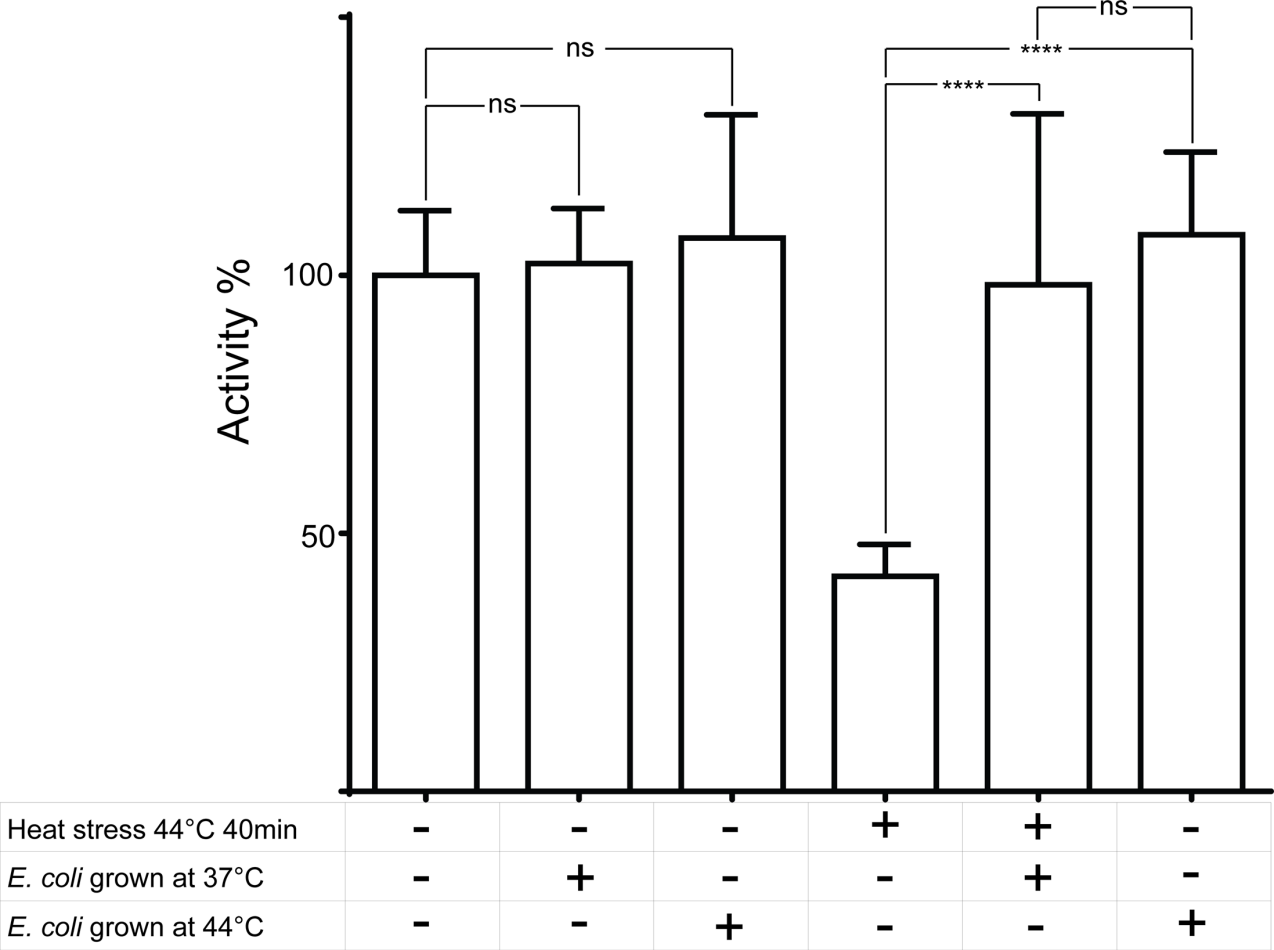
Effect of E. coli total protein extract on the deactivation of CS by high temperature. The activity of citrate synthase was compared before and after temperature stress, between samples with and without the addition of total protein extract from cells grown at 37°C or 42°C as indicated in the grid. The addition of either type of extract significantly protects the activity of CS (shown by t-test). A slight, but not statistically significant, difference was observed between the protective effects of various cell extracts. The height of the bars represents the mean of at least 6 technical repeats, the error flags– 95% CI. The significance of the differences was assessed via an one-way ANOVA.

We also examined if our assay is useful to measure the protein protection capacity of cellular extracts. This can prove useful for comparing how the proteome of a cell or tissue changes upon the effect of a stress factor, facilitating the discovery of novel chaperones. The protective effect of a bacterial protein extract on CS activity was measured and compared to the control reaction where CS is alone (Fig 4). After 40min at 44°C, CS alone loses its activity, while this is not the case when excess of total protein extract is added to the sample. We observe a complete protection of the CS activity by the extracts from both stressed and non-stressed cells. We believe that this is due to constitutively expressed chaperones in *E*. *coli*. Additionally we also tested for non-specific reactions attributed to the enzymes present in the extract, without the addition of purified CS. We found such background reactivity to be comparable to the reactions where all reagents but the enzyme was added (S1 Fig and S1 Dataset). Thus, comparison between the different samples is reasonable and conclusive.

## Discussion

The prevailing pipeline of the identification and characterization of (novel) chaperones relies on homology-based identification of conserved regions and domains from known proteins with such function, and/or experimental testing of proteins upregulated during stress conditions [4,50–52]. While these standard approaches to predicting, identifying and characterizing protein chaperones have brought much success, we address the lack of a quick and reliable method for the (semi) high-throughput *in vitro* screening. Here we report on assays optimized for a plate reader, operating in a semi high-throughput design. We believe that the reported protocols would allow both screening for novel chaperones and gaining a better understanding of the activity of known ones. In the optimization, we have taken into account the possible factors that might compromise the accuracy of the measurement, and took necessary precautions to tackle them. We show that such an assay is capable of pinpointing protective activity of a variety of proteins. Whereas the current protocol applies heat stress, it can be easily adapted to other stress conditions, provided the stress condition can be applied uniformly across all wells, i.e. it does not form a gradient within the plate. Protective effects measured for the classical chaperones used in this assay are comparable with those reported previously (Fig 2D) [53,40–45].

Chaperone activity is an important cellular function of proteins, involved in practically all physiological, and many pathological, processes [12]. Here we established a convenient and time effective tool for the reliable screen of protein activity, which may lead to the identification of novel cellular chaperones. It should not be missed, though, that several cases are known where proteostatic mediators play crucial roles outside the cell, in body fluids, to maintain proteostasis in the intracellular space [54,55]. Known examples are the role of HSP70 in the immune response, and the contribution of such proteins in the prevention of neurodegenerative amyloid diseases. Currently, ELISA-based tests are used to measure the level of plasma chaperones, but they are usually based on lengthy protocols with, at best, semi-quantitative results, only giving information on the approximate amount, but not their activity [56]. While testing of our technique on human samples has not been performed yet, we anticipate it will be of use in clinical diagnostics as an indicative tool.

## Supporting Information

S1 Dataset Raw data of chaperone activity measurements. Dataset includes all raw measurement data obtained throughout the study, separated into sheets according to the type of the measurement.
(XLSX)

S1 Fig Activity of *E*. *coli* whole cell extracts. The background activity of whole cell extracts containing endogenous CS was measured to estimate the cross-reactivity of the extract to the recombinant porcine CS. These background activity measurements were performed for each experiment where whole cell extracts were used (see Materials and Methods section). For each bar, the temperature indicated signifies the temperature at which the cells were grown at before extraction. For every sample we also tested the deactivation of the endogenous CS at high temperature (marked as AS) similarly as it has been performed with the recombinant CS. In every case, the data was normalized to the activity of the recombinant porcine CS, before stress. The data was plotted as bars indicating mean and error flags–the 95%CI.
(TIF)
