# Supplementary figures and images for: A Novel Method for Assessing the Chaperone Activity of Proteins
Source: PLoS One. 2016 Aug 26;11(8):e0161970. doi: 10.1371/journal.pone.0161970 (PMC5001627; doi:10.1371/journal.pone.0161970)

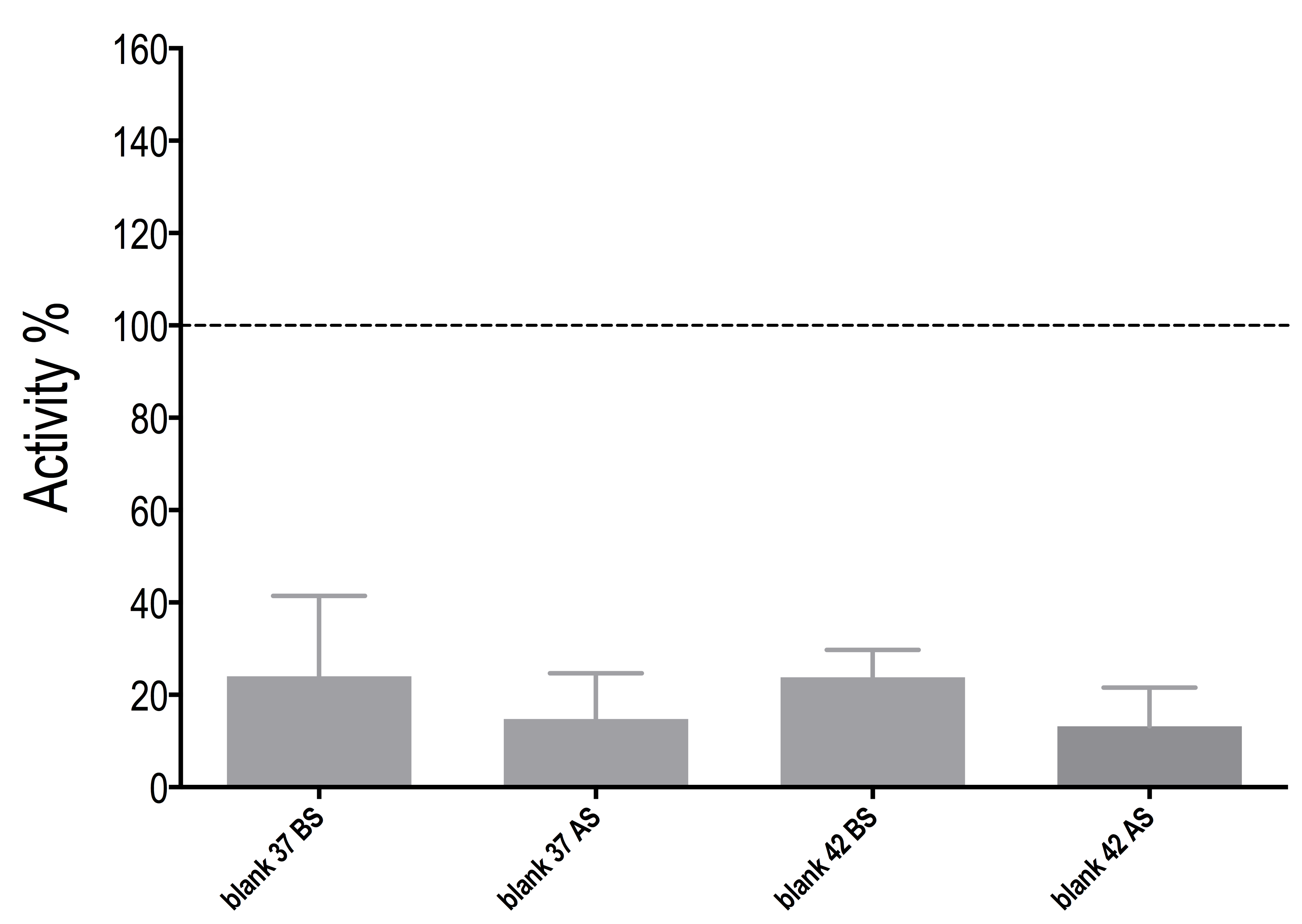

Supplement: S1 Fig — The background activity of whole cell extracts containing endogenous CS was measured to estimate the cross-reactivity of the extract to the recombinant porcine CS. These background activity measurements were performed for each experiment where whole cell extracts were used (see Materials and Methods section). For each bar, the temperature indicated signifies the temperature at which the cells were grown at before extraction. For every sample we also tested the deactivation of the endogenous CS at high temperature (marked as AS) similarly as it has been performed with the recombinant CS. In every case, the data was normalized to the activity of the recombinant porcine CS, before stress. The data was plotted as bars indicating mean and error flags–the 95%CI. (TIF) [file pone.0161970.s002.tif]
